# Diel leaf growth of soybean: a novel method to analyze two-dimensional leaf expansion in high temporal resolution based on a marker tracking approach (Martrack Leaf)

**DOI:** 10.1186/1746-4811-9-30

**Published:** 2013-07-25

**Authors:** Michael Mielewczik, Michael Friedli, Norbert Kirchgessner, Achim Walter

**Affiliations:** 1 ETH Zürich, Institute of Agricultural Sciences, Universitätstr. 2, CH-8092 Zürich, Switzerland

**Keywords:** Marker tracking, Phenotyping, Image analysis, Plant growth, Diel growth, Natural illumination

## Abstract

**Background:**

We present a novel method for quantitative analysis of dicot leaf expansion at high temporal resolution. Image sequences of growing leaves were assessed using a marker tracking algorithm. An important feature of the method is the attachment of dark beads that serve as artificial landmarks to the leaf margin. The beads are mechanically constricted to the focal plane of a camera. Leaf expansion is approximated by the increase in area of the polygon defined by the centers of mass of the beads surrounding the leaf. Fluctuating illumination conditions often pose serious problems for tracking natural structures of a leaf; this problem is circumvented here by the use of the beads.

**Results:**

The new method has been used to assess leaf growth in environmental situations with different illumination conditions that are typical in agricultural and biological experiments: Constant illumination via fluorescent light tubes in a climate chamber, a mix of natural and artificial illumination in a greenhouse and natural illumination of the situation on typical summer days in the field. Typical features of diel (24h) soybean leaf growth patterns were revealed in all three conditions, thereby demonstrating the general applicability of the method. Algorithms are provided to the entire community interested in using such approaches.

**Conclusions:**

The implementation Martrack Leaf presented here is a robust method to investigate diel leaf growth rhythms both under natural and artificial illumination conditions. It will be beneficial for the further elucidation of genotype x environment x management interactions affecting leaf growth processes.

## Background

Plant size and shape is determined by its growth, while growth itself can be influenced by numerous endogenous, genetic, epigenetic and environmental factors. It is well known that leaf growth, many metabolic reactions, physiological processes and elements of regulatory networks show diel (24 h) fluctuations that are partly controlled by the circadian clock [1-12]. Light and temperature beside many other environmental parameters, which can affect that circadian rhythm are the most “potent” and important input factors of circadian entrainment [1,13,14]. Overall, circadian regulation has to be assumed necessary to maintain plant productivity [8]. Growth thus can be considered as the major, integrating output process of plant metabolism, cumulating over time into final leaf size [15,16]. Dynamic fluctuations of growth therefore reflect adjustments of endogenous processes to variations of environmental conditions; their elucidation can be of importance to understand processes of biomass and yield formation [17].

Therefore, it is of vital importance to monitor diel growth patterns as influenced by different environmental conditions [2,18-26], development [27] or by alterations in metabolism [28,29]. Several quantitative methods based on digital image processing and sequence analysis have been developed and applied to study fluctuations in growth of various plant organs such as roots and leaves in recent years [13-15,30-32]. Other non-imaging methods for measurements of growth, such as “classical auxanometers” [33], linear voltage differential transducers (LVDTs) [34,35], rotary resistance transducers (RRTs) [2], direct assessment of plant size or subsequent manual assessment of displacement of markings that have been applied to the organ surface [36,37], exist and have been used both in field and climate chamber experiments. Yet they are limited either to measurements of one-dimensional growth (elongation), are labor intensive needing manual processing steps or do not provide a suitable high temporal or spatial resolution, as do quantitative methods based on image processing, which are thus preferable.

In general the techniques applied can be classified in three groups of image processing approaches [32]: (I) “morphometric”, (II) “optical flow” and (III) “particle / marker tracking” with the two latter methods providing the potential for analysis of spatially differentiating growth or strain rates within the organ if marker or grey value structures within the organ can be followed kinematically.

Morphometric approaches are based on segmentation algorithms calculating projected leaf area and additional shape parameters from analysis of the outline of leaves or leaf rosettes. High-throughput phenotyping methods are typically based on such approaches [38-43]. Even though morphometric image processing has been applied to estimate the projected area and shape of single leaves both in vivo and in studies of harvested leaves [44] it has been found most effective in investigations on a whole plant/shoot level. For investigation of diel growth patterns, morphometric approaches have found only limited usage due to problems arising from leaf motion [32]. Furthermore it is not possible to extract spatial differences in growth rate within the segmented plant organs.

Optical flow based growth estimation is the most powerful method to provide both high temporal and high spatial resolution. Such methods have been applied to study leaf [2,17,45,46], root [47-49] and hypocotyl growth [50]. The movement of structural patterns within the space-time-cube of subsequent images is used to calculate velocity fields of structural elements such as vein intersections, trichomes or ink dots applied on the leaf surface on a subpixel level of accuracy [51], as long as image brightness is constant. Recent improvements allowed to study diel growth patterns of small leaves of the model species *Arabidopsis thaliana*, thereby opening the possibility to even investigate alterations in growth of a wide repertoire of mutant and transgenic plants such as starch deficiency, circadian or photorespiratory mutants [28,29,52]. Yet, optical flow based approaches are sensitive to brightness fluctuations and they require that structural patterns are not moving too fast from one image to the next. Therefore, even though leaves are growing slowly, a huge number of images have to be acquired within short time, as physical structures are not allowed to change position for more than one pixel between consecutive images. This inevitably increases the size of image sequences. Although computer storage capacity and processing speed continuously advance, this problem should not be underestimated. An image sequence showing expansion of a single leaf throughout several days typically needs to comprise several gigabytes (one image per minute) to allow for optical flow based data evaluation. After all calculation, the amount of data typically exceeds 10 gigabytes. With a project typically consisting of dozens of sequences that have been acquired it is obvious that this leads to challenges in data handling, storage, backup and exchange of data even under today’s norms. The problem becomes even potentiated, if it is necessary to increase the spatial resolution, as characteristically higher spatial resolution makes it necessary to also increase the temporal resolution of image acquisition. A second major problem associated with the optical flow approaches is the fact that the so-called ‘brightness change constraint equation’ (BCCE) has to be fulfilled throughout the sequence, which means, that a constant brightness has to be assured [53-56]. Under controlled conditions in climate chambers this is possible throughout day and night by using infrared diode illumination of the scenery and infrared bandpass filters in front of the camera. Yet, under greenhouse conditions and even more in the field, it is nearly impossible to fulfill this intensity requirement due to diurnal fluctuations in illumination.

Marker tracking is a technique of image processing and analysis, in which a discrete number of landmarks is registered initially within the object of interest. The position of these featured landmarks is followed in the consecutive images of an image sequence using pattern matching in the local neighbourhood of these markers. Based on this approach, it is possible to calculate relative growth rate (RGR) with high temporal resolution [57]. Such approaches have been used successfully to study root growth [58-60] by following either artificially applied particles on the root surface or by taking cell walls as markers. In the context of leaf growth though, marker-tracking-based image processing routines only have been applied in a limited number of studies [61,62]. Moreover, in these studies there was no attempt to analyze leaf growth at high temporal resolution or to perform field experiments. In general, marker tracking is a very powerful and robust method in image processing, in which the BCCE requirement does not have to be fulfilled [63]. Therefore, it should be possible in principle to assemble a marker-tracking based approach that allows assessment of diel leaf growth patterns in field experiments.

It was the aim of this study to establish a marker-based approach that allows monitoring of diel leaf growth fluctuation in various illumination conditions, revealing typical features of diel leaf growth patterns that are known from optical flow based approaches, without further consideration of base-tip gradients or other spatial growth differences within the leaf lamina.

## Material and methods

### Growth conditions

Soybean plants (*Glycine max* (L.) Merrill, variety “Gallec”) were grown in plastic pots (10 cm × 10 cm × 10 cm) filled with substrate (“Spezialmischung 209”, RICOTER Erdaufbereitung AG, Aarberg, Switzerland) inside a climate chamber (Conviron, Winnipeg, Canada) under controlled conditions with a 13h/11h light/dark photoperiod: light intensity 580 ± 75 μmol PAR m^-2^ s^-1^; average temperature of 24°C (day) and 20°C (night); relative humidity 60% (day and night). The climate chamber was equipped with a 2:1 mixtures of fluorescent lamps of two types (Master TL5 HO 54W/840, Koninklijke Philips Electronics N.V., Eindhoven, the Netherlands and FHO54W/T5/GRO, Havells Sylvania Europe Ltd, London, UK).

Additional soybean plants (variety “Amphor”) were grown in a greenhouse and in the field of the research station for plant science of ETH Zurich in Lindau-Eschikon. Soybean plants in the greenhouse were grown in plastic pots as described above, filled with substrate (“Spezialmischung 209/09-047”, RICOTER Erdaufbereitung AG, Aarberg, Switzerland). The plants were kept under standard greenhouse conditions and were watered on a regular basis. In the field, plants were sown in small plots (6.5 m length, 1.5 m width, 18 cm row width, 60 seeds/m^2^).

### Mechanical leaf fixation and preparation of image acquisition

**Figure 1:**
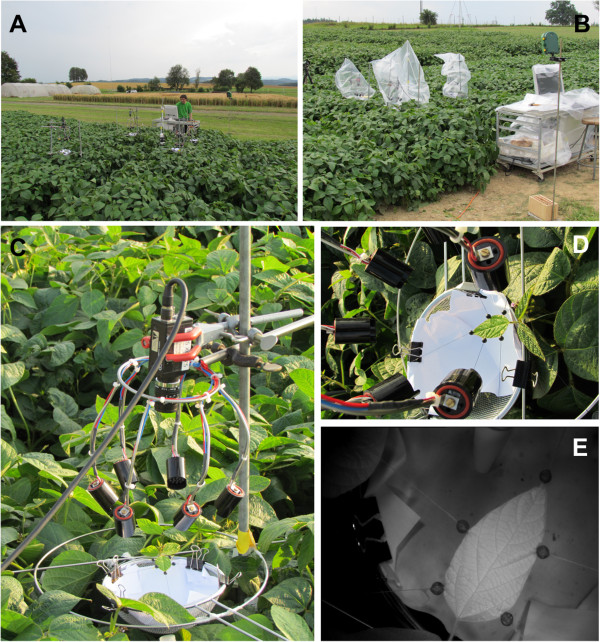
**Setup used in the field. (A)** Overview of the soybean field and setup; **(B)** Setup wrapped in plastic bags due to rain (measurement stopped); **(C)** Close-up view of the setup with infrared camera on top, infrared diodes and a soybean leaf fixed with strings glued to the leaf with attached weights; **(D)** Close-up view of the fixed soybean leaf with attached black beads; **(E)** Original image of a soybean leaf in the field taken with an infrared camera.

**Figure 2:**
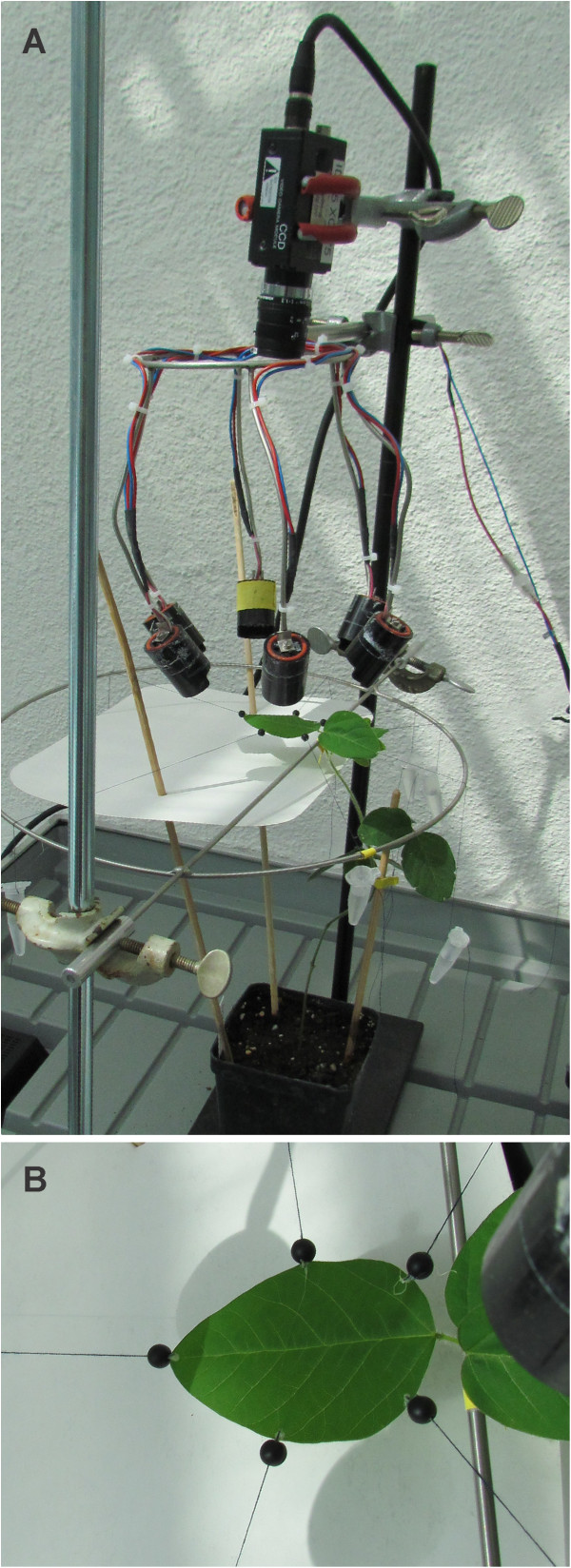
**Setup used in the greenhouse. (A)** Camera, infrared diodes and a soybean leaf fixed in a metal frame; **(B)** Close-up view of a fixed soybean leaf with attached black beads and threads.

**Figure 3:**
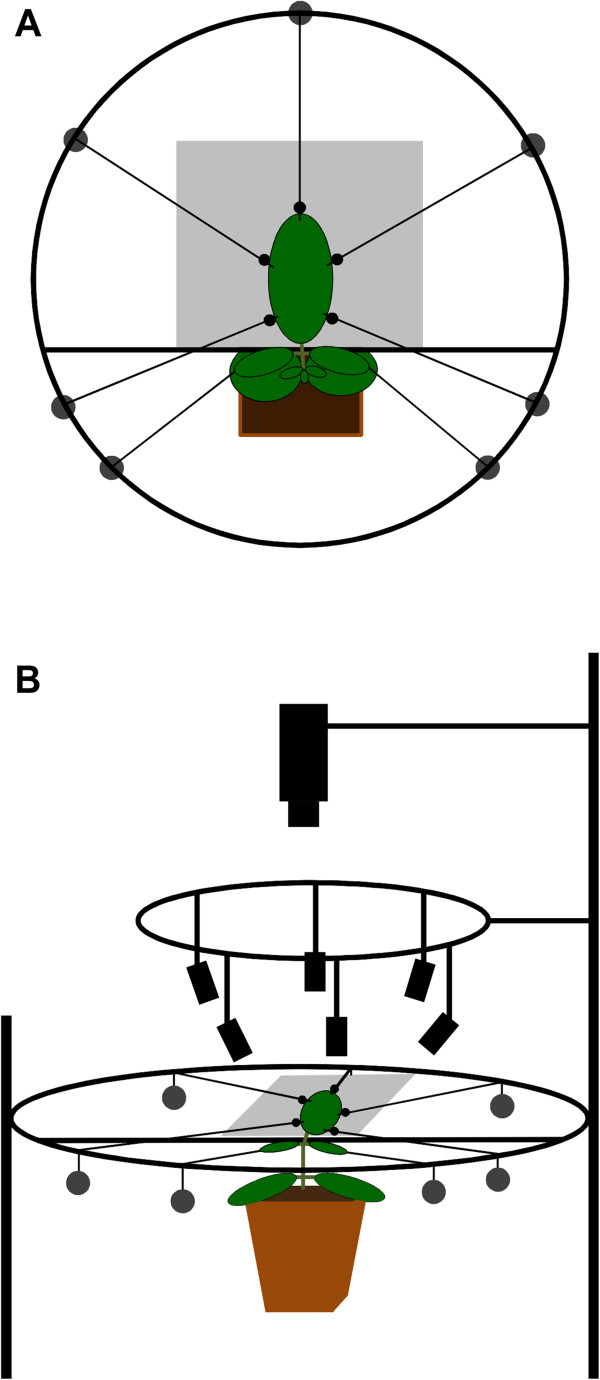
**Principle sketch of setup. (A)** Top view of soybean leaf fixed with strings in a metal frame with marker beads attached at the leaf margin, the artificial background is shown in gray; **(B)** Whole setup in top side view with camera, infrared LED clusters and fixed soybean leaf.

One growing leaf of every investigated plant was fixed in the focal plane of a top mounted camera placed above the abaxial leaf surface using 5 small weights of 2.5 to 9 g attached with strings and glue (Pattex® KRAFTKLEBER Classic, Henkel AG & Co. KGaA, Düsseldorf, Germany) to the leaf surface (Figures 1 and 2) [2,28,29,49]. Small leaves were fixed using small weights; preliminary experiments showed that the weights did not affect final leaf size or shape. Weights were hung over a circular metal frame around the leaf. An additional weight was used as counterforce attached at the opposite side of the shoot to avoid unwanted movements of the plants. Additionally, parafilm was used to fix the leaf at its base to a thin metal bar in the ring without hurting the plant, thereby assuring that stem elongation did not lift parts of the leaf above the focal plane of the camera during acquisition of the image sequence. Black plastic beads (5 mm diameter) were glued to the strings at the leaf border to provide artificial landmarks that allowed registration of marker movements, see sketch in Figure 3.

To allow continuous measurement of plant growth during night and day, a metal ring with 6 infrared LED clusters (940 nm) was used as illumination source (see [28,29]). Usage of infrared LEDs has two major advantages: (1) leaf growth and plant metabolism are not affected by near-infrared light beyond 800 nm and (2) as leaves diffusely reflect more light in the infrared region of the spectrum, contrast between leaves and background is enhanced while specular reflexes can be avoided.

### Image acquisition

Monitoring and analysis of growth in greenhouses and in the field was performed using a standard progressive monochrome CCD camera (XC-55, Sony Corporation, Tokyo, Japan) linked to a personal computer (PC). Images of the growing leaf were acquired every 90 seconds with a resolution of 640 × 480 pixels. The camera was equipped with a lens (H1214-M, 12 mm 1:1.2, Pentax Ricoh Imaging Co., Ltd, Tokyo, Japan) and a narrow bandpass interference infrared filter (940 nm, Edmund Optics Ltd, York, UK) to improve overall image quality and to allow continuous measurement under artificial near-infrared illumination during night and day with fixed camera settings. Automatic gain correction (AGC) was activated for image acquisition under field conditions. Image acquisition in the near infrared region of the spectrum is generally possible in this setup, as standard CCD cameras typically are sensitive up to a wavelength of 1100nm if no additional hot-mirror or filter is placed inside the camera. Thus, the overall setup is highly comparable to that of the so-called ‘digital image sequence processing (DISP) setup’ [30,32] that has been used frequently to monitor diel leaf growth patterns via an optical flow based algorithm. Image acquisition in the field was performed on several consecutive days without rain to avoid any hardware defects. No additional shelter was applied.

Image acquisition in the climate chamber was conducted with monochrome CMOS cameras (DMK 23GP031, The Imaging Source Europe GmbH, 28215 Bremen, Germany). Each camera was equipped with a lens (C2514-M, 25 mm 1:1.4, Pentax Ricoh Imaging Co., Ltd, Tokyo, Japan) and a narrow bandpass interference infrared filter (940 nm, Edmund Optics Ltd, York, UK). The cameras were linked via Ethernet cables to a switch (SG300-10P, Cisco Systems Inc., San Jose, USA) and this in turn linked to a router (Cable/DSL Web Safe Router RP G14 v4, NETGEAR Inc., San Jose, USA) and to a PC. Images were acquired every 90 seconds with a resolution of maximally 2592 × 1944 pixels and the images were stored directly from the software “IC Capture” (The Imaging Source Europe GmbH, 28215 Bremen, Germany) to a PC. By using a switch, several cameras were operated simultaneously with only one PC. Another advantage of these cameras was that they were powered over Ethernet so no additional power supply was needed.

### Algorithm and software for optimized leaf growth analysis

**Figure 4:**
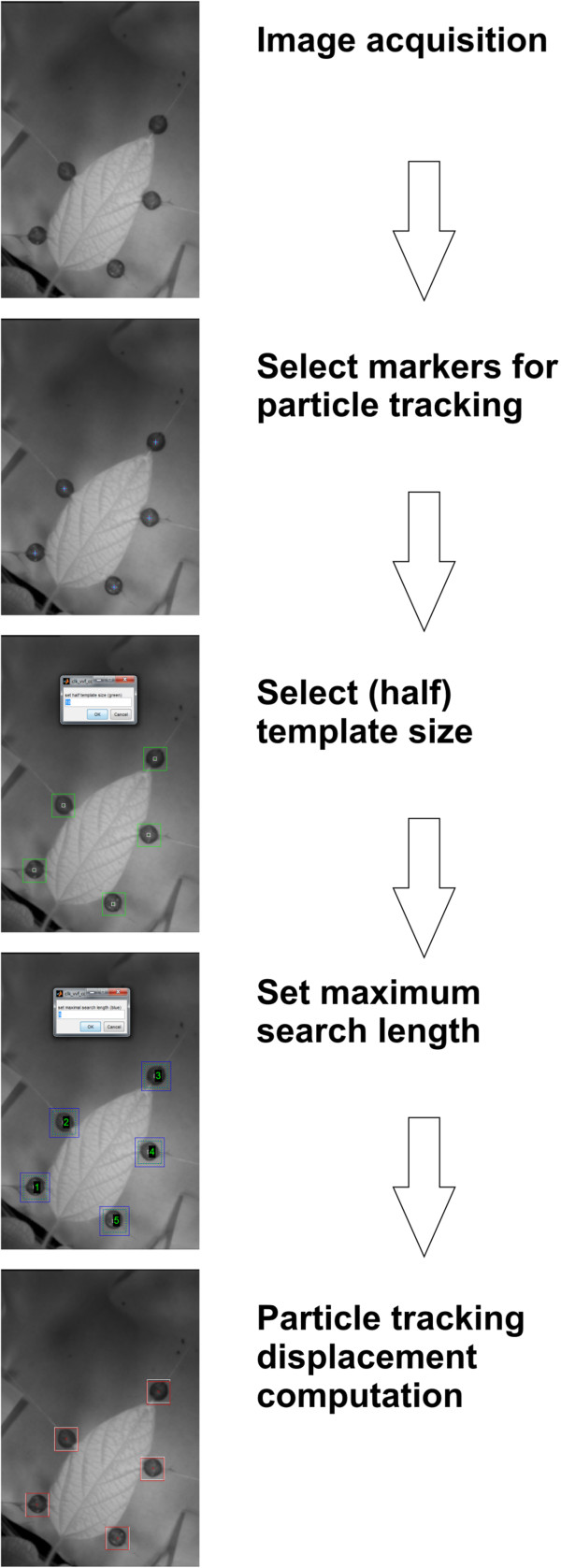
**Analysis of image sequence.** (**1**) Images need to be taken. (**2**) Centre of each black bead must be selected. (**3**) The template size around the black bead has to be chosen. (**4**) The maximum search length of the template must be given. (**5**) In each image the positions of the five black beads are automatically tracked. (**6**) The area of the polygon defined by the positions of the 5 black beads is computed for each image. (**7**) Relative growth rate is calculated from the increase in area. (For a detailed step by step guide on Martrack Leaf consult the manual (see Additional file 1).

The algorithm for marker tracking was implemented in Matlab 7.12 (The Mathworks, Natick, MA, USA). Template sizes of 23 × 23 pixels up to 85 × 85 pixels depending on image resolution were tracked in the local neighborhood with a search length of 6 pixels and larger. These parameters can be freely adjusted using a graphical user interface. The small black beads attached to the leaf margin were used as artificial landmarks and were selected by clicking on them in the initial image of the image sequence in the graphical user interface (Figure 4).

Based on the calculated area it is possible to calculate relative growth rate (RGR) of the enclosed pentagon for every frame of the image sequence by using the following equation (A_1_ area at image (frame) number f_1_):

$$\mathrm{RGR}\;\left(\mathrm{in}\%\mathrm{per}\;\mathrm{frame}\right)=\frac{\left(\ln A_{2}-\ln A_{1}\right)}{f_{2}-f_{1}}\;100$$

To calculate RGR in percent per hour for every frame, a time correction factor f_c_ corresponding to the number of images acquired during every hour was applied:

$$\mathrm{RGR}\;\left(\mathrm{in}\%\mathrm{per}\;\mathrm{hour}\right)=\mathrm{RGR}\;\left(\%\mathrm{per}\;\mathrm{frame}\right){\;f}_{c}$$

As images were acquired every 90 seconds, a time correction factor of 40 was used throughout the experiment.

**Figure 5:**
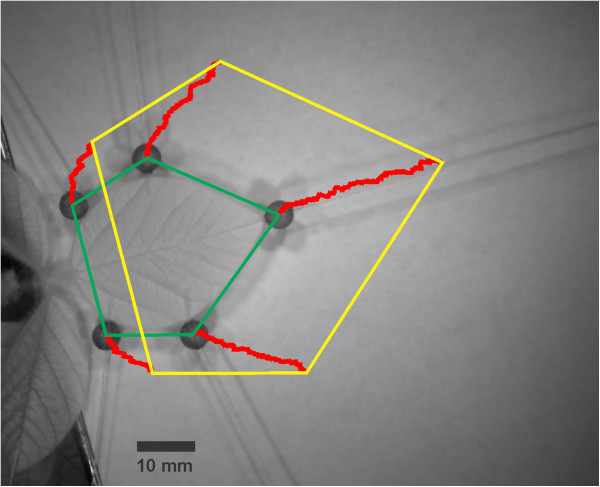
**Illustration of the growth of a soybean leaf in a climate chamber.** The five red lines are overlaid to the first image of a sequence. They indicate the path of the center of every single black bead tracked over a period of 86.5 hours with Martrack Leaf. The green pentagon shows the leaf area at beginning of the measurement whereas the yellow pentagon denotes the area at the end of the measurement.

The graphical user interface (GUI) implemented in Matlab was kept as simple as possible with processing scheme implemented as shown in Figure 4. Initially the recorded image sequence is opened and in the first frame of the sequence the center of each bead is selected separately by mouse-clicks. Afterwards, a template-size around the black beads has to be defined. In the following step, a search length has to be assigned for the neighborhood in which the bead has to be found in consecutively following images (frames). The calculation is then started and every black bead is tracked throughout the whole sequence. At the end of this process, the path of the center of every tracked bead throughout the sequence is displayed with a red line in the starting image (Figure 5).

The block matching algorithm determines the best position of each template in the current image by normalized cross-correlation (CC) [63].

$${}_{x,y\in S}\mathrm{CC}\left(x,y\right)=\frac{\sum_{m,n}\left(i\left(x+m,y+n\right)-I_{\mathrm{xy}}\right)*\left(t\left(m,n\right)-T\right)}{\sqrt{\sum_{m,n}{\left(i\left(x+m,y+n\right)-I_{\mathrm{xy}}\right)}^{2}*\sum_{m,n}{\left(t\left(m,n\right)-T\right)}^{2}}}$$

Where S is the search area, x,y are the upper left coordinates of the image area which is compared to the template, i is the image. I_xy_ is the mean gray value of the image area which is matched to the template t, and T is the mean gray value of t. The coordinates within the matched image area, both of i and the template t, are called m and n.

Template size and search area are adjustable to the image material and temporal sampling of the scene. For each template the CC is calculated around the position in the last investigated image in the chosen search area. The best position is localized by the maximal CC value CC_max_. If CC_max_ is lower than 0.7 the template is regarded as not found, which ensures a high quality of template positions.

Subpixel accuracy of template positions is achieved by using a quadratic interpolator for the cross-correlation [64]. The maximum of the quadratic polynomial defined by CC_max_ and its neighboring CC values in x-direction therefore gives the subpixel position in x-direction; the subpixel position in y-direction is determined accordingly.

For each frame, the bead positions define a polygon, which approximates the leaf area. The area of the polygon is calculated with the Gauß-formula for trapezes:

$$A=0.5\overset{n}{\underset{i=1}{\sum }}y_{i+1}x_{i}-y_{i}x_{i+1}$$

Where A is the polygon area, x,y are the coordinates of the n corner points of the polygon. Indices are regarded modulo n, which means x_n+1_=x_1._

Martrack Leaf is provided online compiled for different operating systems (for detailed manual see Additional file 1, linux see Additional file 2, Mac see Additional file 3 and Windows 64-bit see Additional file 4). In order to execute Martrack Leaf, the Matlab Compiler Runtime (MCR; Vers. R2012a 64-Bit) (The Mathworks, Natick, MA, USA) needs to be installed on the user machine (download at http://www.mathworks.com/products/compiler/mcr/). Files with extension .fig are figures in a Matlab- specific format, they can be displayed, printed and saved using the provided executable showfigure.exe (included in the Additional files for each operating system).

## Results

In preliminary experiments, different wooden and plastic beads from local crafts stores were tested for their optical properties. Most of the black beads tested fulfilled the requirement to show a low reflectance in the near-infrared range. Therefore, it was easy to differentiate beads from the leaves and leaf margins. Nevertheless, soil and non-soil shadowed backgrounds also often appeared very dark, which made it impossible to maintain contrast differences between beads and background under many conditions.

**Figure 6:**
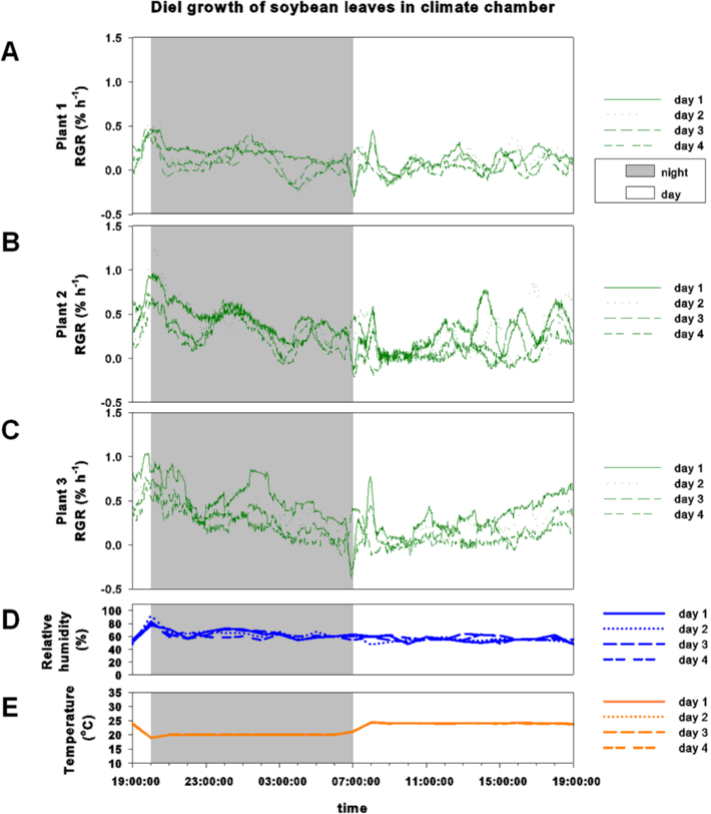
**Diel growth pattern of soybean leaves in the climate chamber. (A, B, C)** Relative growth rates (RGR) of three soybean leaves from day 1 to day 4. **(D)** Relative humidity (%) and **(E)** temperature (°C) throughout the measurements.

It was therefore beneficial to introduce a bright background below the leaf, thereby increasing contrast between beads and background (see Figures 1 D+E and 2). Experiments performed in climate chambers showed clearly oscillating temporal leaf growth patterns (see Figure 6) RGR increased during the day, reaching a maximum at day-night-transition. At night, RGR declined again. Short-term fluctuations of RGR were present, but the overall diel pattern remained constant throughout several days.

**Figure 7:**
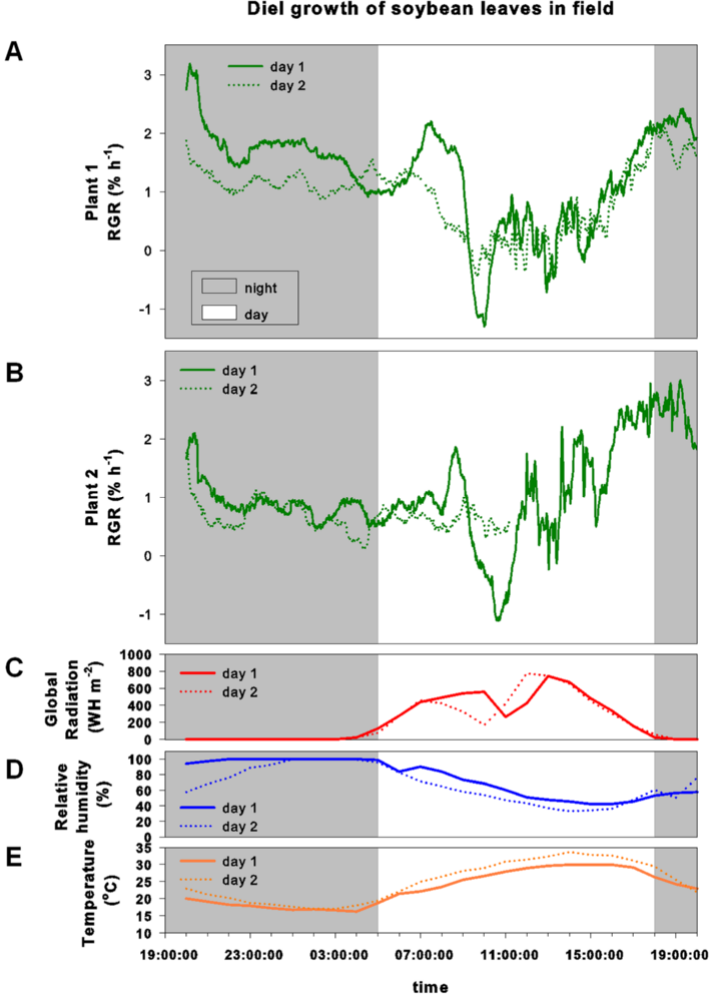
**Diel growth pattern of soybean leaves in the field. (A, B)** Relative growth rates (RGR) of two soybean leaves on day 1 and day 2; **(C)** global radiation (Whm-2); **(D)** relative humidity (%) and **(E)** temperature (°C) throughout two days in the field.

In the field, similar diel leaf growth patterns were obtained (Figure 7). At noon though, plants grown in the field showed a pronounced, transient drop in their RGR. Fixation of leaves in the field worked well, and movement of leaves was prevented by leaf fixation even when relatively strong gusts of wind were present. In a period of dry weather, it was therefore possible to monitor leaf growth on three consecutive days without rain. Since the setup and outdoor computer installation was not waterproof, care was taken to protect it from eventually upcoming rain if necessary (Figure 1B).

**Figure 8:**
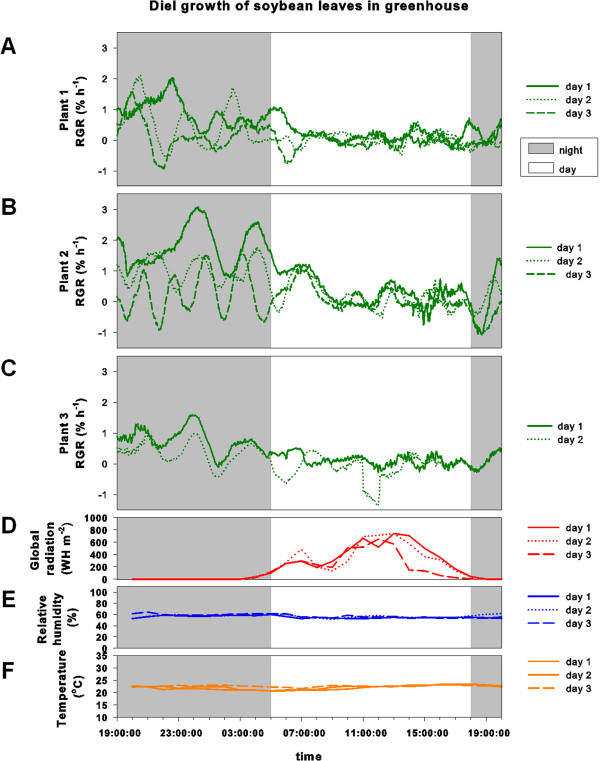
**Diel growth pattern of soybean leaves in the greenhouse. (A, B, C)** Relative growth rates (RGR) in % per hour of three soybean leaves for day 1-3 **(A,B)** and day 1 and 2 **(C)**; **(D)** Global radiation (Whm-2); **(E)** relative humidity (%) and (F) temperature (°C) for corresponding days.

In greenhouse experiments, maximal growth was also observed at night and the overall growth patterns were similar to the patterns obtained in the climate chambers and in the field (Figure 8). Yet, under these conditions, secondary fluctuations of leaf RGR occurred at night with a phase length of two to three hours.

Light intensity in the climate chamber was kept constant at around 580 ± 75 μmol PAR m^-2^s^-1^ during the light period and it was completely dark during night (data not shown). In the field, global radiation increased continuously beginning at around 4 am and reached its peak at noon but then started again to decline towards dusk (Figure 7). At both measuring days, a temporary drop in global radiation before noon was observed in the field. In the greenhouse, a similar pattern as seen in the field occurred with a drop in global radiation in the middle of the morning (Figure 8).

Relative humidity in the climate chamber was kept constant at 60% with no strong fluctuations during all four days (Figure 6). In the field, relative humidity was highest during night and decreased continuously during the day reaching its minimum value in mid-afternoon (Figure 7). In greenhouse experiments, relative humidity was kept constant at around 60%, comparable to climate chamber experiments (Figure 8).

Temperature in the climate chamber was kept at 24°C during the light period and at 20°C during night (Figure 6). Temperature at the field site fluctuated more severely compared to greenhouse and climate chamber conditions, reaching a maximum in mid-afternoon (Figure 7). In greenhouse experiments temperature showed no strong fluctuations and was kept at roughly 22°C (Figure 8).

## Discussion

### Diel growth patterns

Image analysis of soybean leaf growth based on the Martrack Leaf algorithm was shown to be robust under indoor and outdoor conditions. The basic pattern of diel leaf growth was comparable to patterns described before, which were analyzed by optical flow based approaches [65]. Based on the few data series acquired here, it is not possible to conclude on the statistical significance of the differences of the diel growth cycles obtained under the different illumination conditions. The focus of the manuscript is to show that the method produces meaningful results even under very different illumination conditions. Nevertheless, it is important to point out that similarities and differences between treatments with respect to the resulting diel leaf growth cycles are physiologically reasonable. The observed midday leaf growth depression in the field, for example, might be a short-term stress reaction, which could have been linked to a general water vapor deficit, or to adaptions in transpiration rates under full sun exposure. It is well-known that short-term alterations in turgor pressure lead to short-term growth peaks or troughs [17,30]. At this time of the day, also global irradiation decreased transiently, suggesting another possible reason for the deviation of leaf growth in the field from the smoother pattern observed in the climate chamber. This has to be investigated in more detail in future studies. In the greenhouse, growth rates were rather low and highest growth activity was observed during the night. Again, the overall diel growth pattern was comparable to the pattern described previously [65] and it did not show a direct relation to air temperature but seems to be rather dominated by endogenous control mechanisms. It is principally possible, that those differences are related to differences in leaf and plant age: Plants investigated in climate chamber, greenhouse and in the field experienced different environmental conditions throughout their entire development. Therefore, it was not possible to compare growth of leaves of identical plant developmental stages. To date, there is no indication in literature, how severely differences in plant development might potentially affect diel leaf growth patterns.

### Comparison to other methods

Martrack Leaf is more robust than optical flow based approaches and provides higher experimental versatility compared to morphometric analyses or to mechanical analyses of leaf elongation growth – such as linear variable displacement transducer (LVDT) or rotary resistance transducer (RRT) approaches (Additional files 5 and 6: Tables S1 and S2). Compared to optical flow analysis, marker tracking allows for larger movements of the tracked structures. Markers can be tracked in consecutive images as long as they move less than the selected search length. To circumvent confounding of different markers (beads) the search-length should be chosen smaller than the closest distance between two markers.

In optical flow analysis, movement of structures must not exceed one pixel per frame. Martrack Leaf allows image analysis with fewer images acquired within 24 h to reveal basic diel leaf growth patterns compared to optical flow based procedures such as DISP. Moreover, wind-induced shifts of the leaf from one image to the next do not pose a severe problem to Martrack Leaf.

Another reason, why optical flow based approaches do not provide high chances for successful leaf growth analyses in the field is the requirement of the BCCE. Only under special circumstances, such as practically cloudless days [17], image brightness changes are slow enough to provide constant brightness throughout a large stretch of the image sequence. To avoid brightness changes within image sequences, automatic adjustments to image brightness are typically used in industrial and consumer image acquisition. This can be realized for example by Automatic Gain Correction (AGC) which can be applied to either the complete image or to a selected region of an image or object. Yet for growth analysis based on grey value intensities, such as in the optical flow based DISP method, AGC usage causes additional problems and is thus typically avoided by using manually fixed set values: In typical image sequences of growing leaves, brightness changes often occur heterogeneously for example in the form of shadows from neighboring leaves or technical structures that travel slowly through the image sequence or that increase in size. These brightness gradients can disturb the quality of an image sequence severely and they can lead to unwanted artifacts in the calculation of RGR. If these artifacts are corrected for automatically, the relation between brightness of neighboring structures is shifted, which leads to the situation that those structures cannot be followed correctly. Biases can also be caused by changes in the reflectivity of the background: Soil for example normally shows a very low reflectivity in the near infrared part of the spectrum but its reflectivity increases with reduced water content.

Optical flow based methods provide advantages with respect to the spatial differentiation of growth rates within the analyzed leaf and they often do not require application of marks to the leaf surface. It also has to be pointed out that brightness changes can lead to problems also in marker tracking approaches since they also hamper marker recognition: Tracking of the marker structures in Martrack Leaf is based on block matching algorithms. Block matching algorithms are frequently used in motion estimation especially for video compression [66-68] or other applications such as the evaluation of microscopic images [69]. As the block matching algorithm compares image patches, any change of the compared patches due to occurring or moving shadows lowers the accuracy of the position determination. This could be circumvented by a template matching algorithm which will be implemented in further work. Furthermore, the chosen template size and search length as well as the image resolution of the sequence can have an effect on the analysis by the algorithm. Thus, the template size should be chosen in a way that allows for the bead to fit inside the template, but the template size should not be selected markedly larger than the bead. The search length needs to be chosen in a way that the bead can be tracked during the whole sequence. Block matching algorithms could also be implemented in optical-flow based techniques, but this is cumbersome and would increase computing time and would not give any advantage as long, as the growth of whole organs is regarded.

### Issues related to storage and image processing times

One typical difference in practical handling of the different methods used for diel leaf growth analysis is related to the amount of storage and processing and evaluation time necessary for calculations and long term backup. Typically RRTs provide the fewest problems in this respect. Acquired data stacks are very small, even if acquisition is performed in very high temporal resolution. In contrast the DISP method produces enormous stacks of data for every plant investigated. Image sequences of growing leaves acquired for DISP analysis over several days characteristically have a size of 1 to 2 GB depending on the selected image resolution, bit depth, time between frames and the overall duration of image acquisition. If higher temporal resolution is necessary (since leaves are otherwise growing too rapidly to fulfill the requirement of a maximal velocity of 1 pixel per frame), or if plants are monitored for many consecutive days, sequences can easily excess sizes of 2 GB. Image sequences acquired for DISP are very big and their evaluation requires creating of several other files that contain the information on velocities of all image pixels in x- and y-direction and on quality estimations. These additional files exceed the size of the original image sequences. Thus, overall size of image sequences for each investigated plant/leaf typically lies in the range of 5 to 8 GB. An experimental design with several replicates under different environmental conditions or mutant lines easily can be in the range of 100 to 1000 GB if standard camera resolutions (640 × 480 to 800 × 600 pixels) are selected.

## Conclusion

Robust tracking based methods such as Martrack Leaf will be beneficial for the further elucidation of genotype × environment × management interactions affecting leaf growth processes. Thereby, they will be able to play an important role both for the elucidation of processes controlling leaf growth and for an improved understanding of growth reaction to the variation of environmental parameters alike.

## Competing interests

The authors have no competing interests to declare.

## Authors’ contributions

NK designed the measurement strategy and implemented Martrack Leaf in Matlab. Pre-experimental beta testing of the algorithm implementation was performed by MM, MF and NK. MM drafted the manuscript with help and contributions by MF, NK and AW. MM, MF and AW designed and performed experiments for leaf growth analysis in field, greenhouse and climate chambers. Growth analysis of soybean leaves using Martrack Leaf was performed by MM and MF with figure plates and eventually created and layouted using Sigma Plot 12 and Corel Draw X4. All authors read and approved the final manuscript.

## Supplementary Material

Additional file 1 Software manual for Martrack. Click here for file

Additional file 2 Software package Martrack for Linux. Click here for file

Additional file 3 Software package Martrack for Mac. Click here for file

Additional file 4 Software package Martrack for Windows. Click here for file

Additional file 5: Table S1 Overview of features related to data processing in methods to calculate diel relative growth rates (RGR) of leaves. Click here for file

Additional file 6: Table S2 Overview of features of the mechanical setup and preparatory steps for data acquisition to monitor diel relative growth rates (RGR) of leaves. Click here for file
