# Supplementary material for: Diel leaf growth of soybean: a novel method to analyze two-dimensional leaf expansion in high temporal resolution based on a marker tracking approach (Martrack Leaf)
Source: Plant Methods. 2013 Jul 25;9:30. doi: 10.1186/1746-4811-9-30 (PMC3750653; doi:10.1186/1746-4811-9-30)
Supplement: Additional file 1 — Software manual for Martrack. [file 1746-4811-9-30-S1.pdf]

Manual for *Martrack_Leaf0_1*

1. Execute the Matlab compiler runtime (MCR). (A shell window providing the MCR environment is opened.)

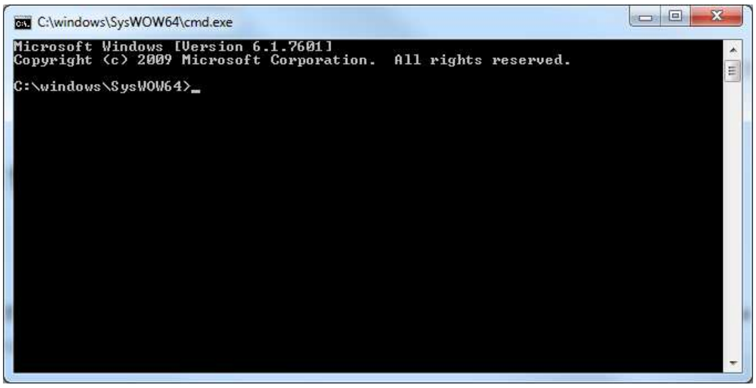

2. Drag and drop the Martrack_Leaf0_1.exe into the shell window, activate it by mouse click, and press "enter".

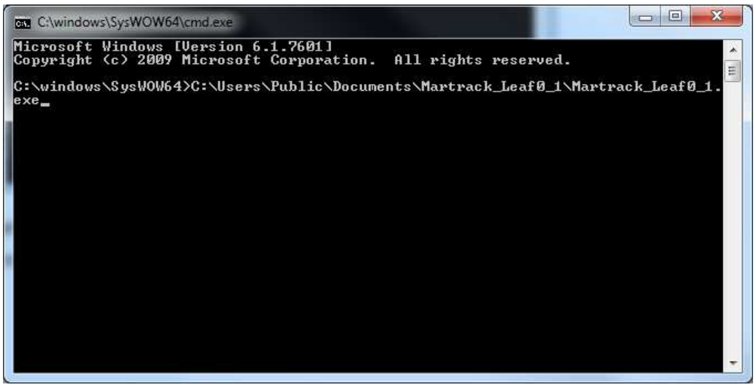

3. A new window opens and asks, if you want to save each image with the tracked regions. Those images show the tracked bead position and are saved as a TIFF stack. (If you chose yes, the evaluation will take noticeably longer.)

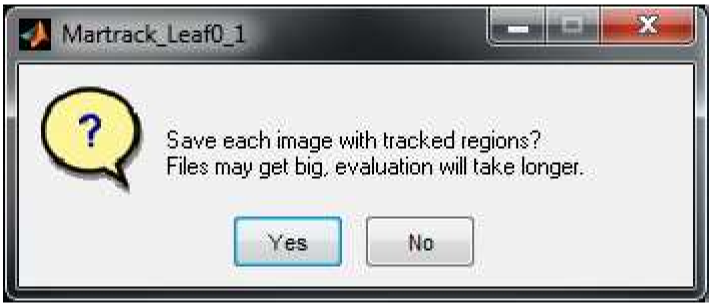

4. A new window opens and you have to choose the first image of your image-sequence.

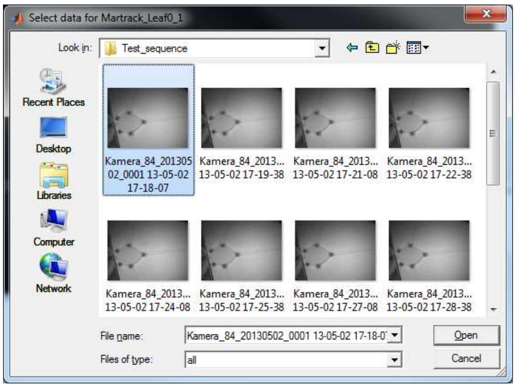

5. In the next window the first image of the sequence is opened and you must select the center of each bead by mouse-click. Backspaces remove the last marked position. Double-clicks finish the bead selection.

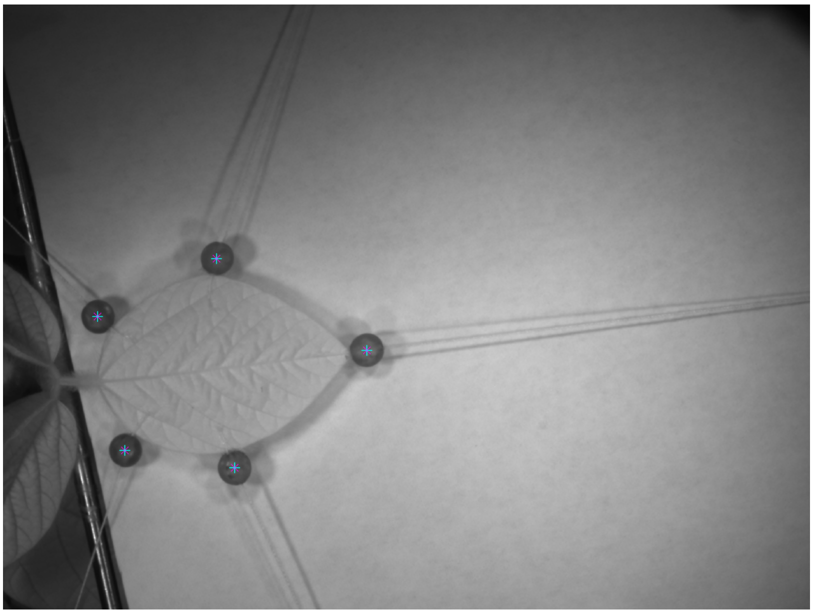

6. After each bead had been selected, the template size around each bead has to be defined for all beads. After typing a new template size, it can be visualized as image overlay by clicking “Try”. As soon as the template size fits, click “Ok” to continue.

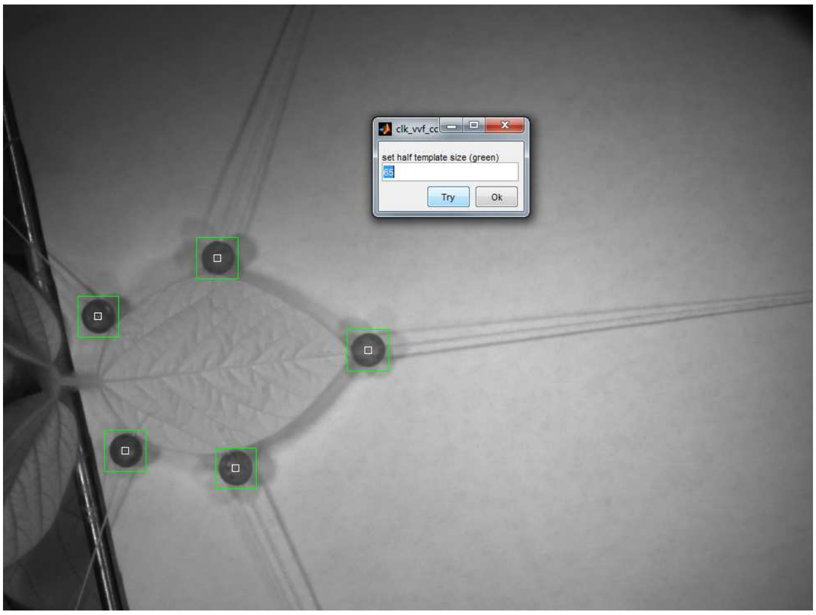

7. In the next window you have to define the search length. After typing a new search length, the corresponding search area can be visualized as image overlay by clicking “Try”. As soon as the search area fits, click “Ok” to continue.

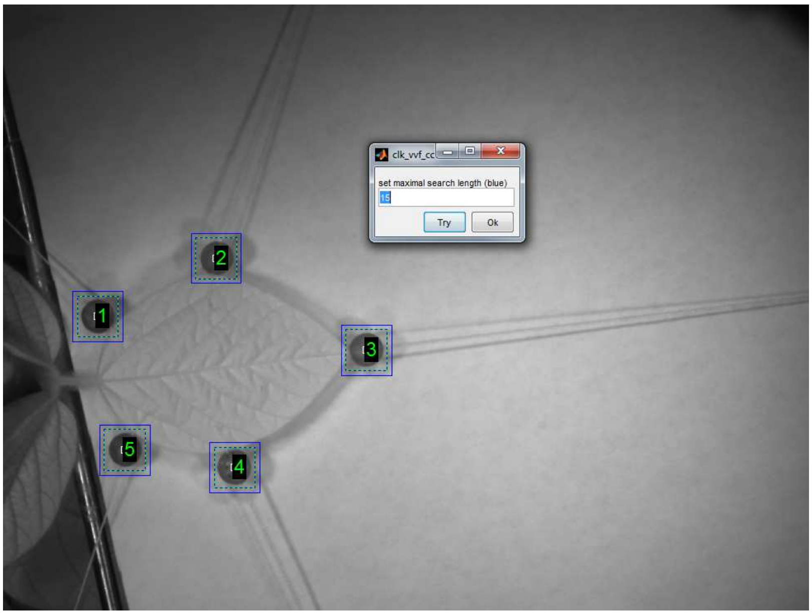

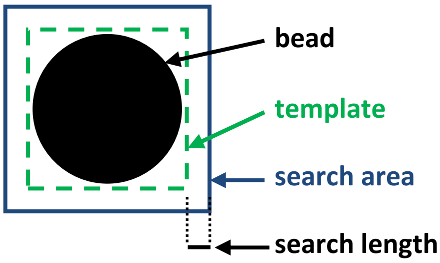

8. Now the calculation starts, and every bead is tracked throughout the whole sequence.

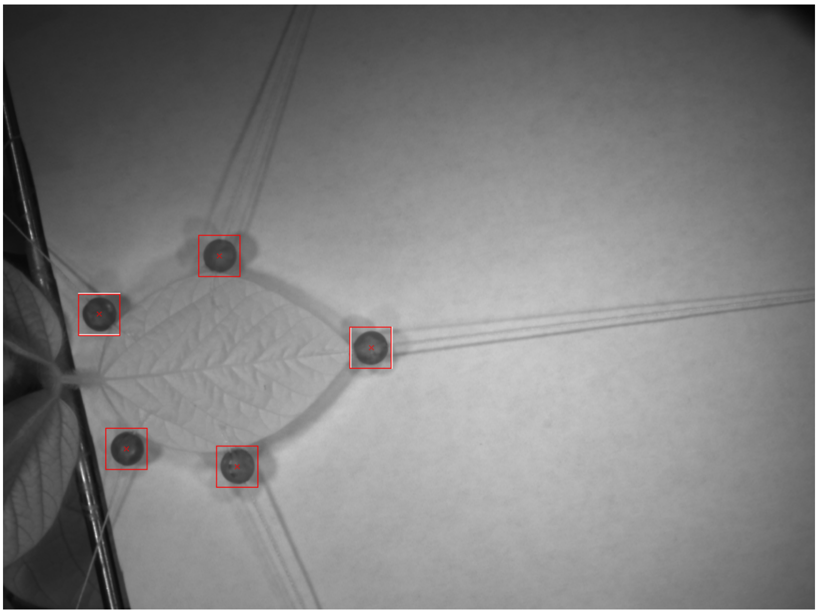

9. At the end of this process a window with the message “Displacement evaluation done, click to continue” opens.

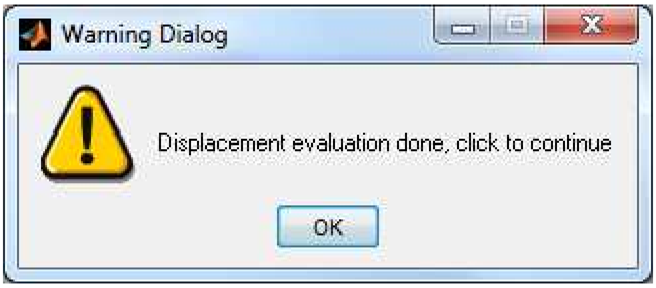

10. After clicking “Ok” figures with area/time-plots and the relative growth rate (RGR) over time are displayed. In the subfolder “VVF_eth” you can find different figures and an excel-file (columns are commented inside the file). Files with extension .fig can be opened and viewed with the provided tool showfigure.exe (see Supplement 3).
