# Supplementary material for: Diel leaf growth of soybean: a novel method to analyze two-dimensional leaf expansion in high temporal resolution based on a marker tracking approach (Martrack Leaf)
Source: Plant Methods. 2013 Jul 25;9:30. doi: 10.1186/1746-4811-9-30 (PMC3750653; doi:10.1186/1746-4811-9-30)
Supplement: Additional file 5: Table S1 — Overview of features related to data processing in methods to calculate diel relative growth rates (RGR) of leaves. [file 1746-4811-9-30-S5.pdf]

Table 1: Overview of features related to data processing in methods to calculate diel relative growth rates (RGR) of leaves. Limitations of the different setups are underlain in red, optimal usability is highlighted in green:

	Morphometric leaf growth	Marker tracking leaf growth analysis	Optical flow based analysis (DISP)	RRTs / LVDTs
Image analysis based algorithm:	Threshold segmentation	Tracking of artificial landmarks	Structure tensor and optical flow	-
Temporal Resolution	high	high	high	high
Spatial Resolution			high	
Calculation of local RGRs in regions of interest within the leaf	no	no	yes	No
Analysis of leaf elongation	yes	no[1]	no[2]	yes
Analysis of leaf width growth	yes	no[1]	no[2]	no
Tracking of leaf elongation biased by petiole growth	no	no	no[3]	yes
Susceptible for changes in brightness	no	no	yes	no
Susceptible for shadows	no	yes	yes	no
Possible to use in field / greenhouse	yes[4]	yes	no	yes
Needs high resolution images	yes (for best quality)	yes (for best quality)	yes (for best quality)[5]	
If temporal resolution can be decreased, image acquisition steps can be skipped to reduce size of image sequences and calculation time	yes	yes	no, or at least highly limited	
Susceptibility to lossy image and video compression	low	low	high	
Optimal to analyze dicot or monocot leaf expansion	dicot	dicot	dicot	monocot and dicot
Necessary storage capacity (including calculation results)	very low to high	medium	high to very high	low
Computing time	low	medium	high	very low

[1] Dependent on beads arrangement (limited)
[2] Theoretically, but not used so far
[3] Growth of petiole is not allowed to move the leaf too fast

[4] Robust against illumination changes as seen in field or greenhouses, yet analysis of leaf growth does not allow movement caused for example by wind. As long as those can be prevented (for example in open-top-chambers), outdoor analysis is possible.

[5] Higher resolutions might give better RGR quality, but number of acquisitions over time must be increased and adjusted, too. For best results, displacements should not exceed one pixel per frame.
