# Supplementary material for: Diel leaf growth of soybean: a novel method to analyze two-dimensional leaf expansion in high temporal resolution based on a marker tracking approach (Martrack Leaf)
Source: Plant Methods. 2013 Jul 25;9:30. doi: 10.1186/1746-4811-9-30 (PMC3750653; doi:10.1186/1746-4811-9-30)
Supplement: Additional file 6: Table S2 — Overview of features of the mechanical setup and preparatory steps for data acquisition to monitor diel relative growth rates (RGR) of leaves. [file 1746-4811-9-30-S6.pdf]

Table 2: Overview of features of the mechanical setup and preparatory steps for data acquisition to monitor diel relative growth rates (RGR) of leaves. Generally related problems and most time consuming steps are underlain red. Similar adjustment steps that are less time consuming or less problematic are highlighted in green.

	Morphometric leaf growth	Marker tracking leaf growth analysis	Optical flow based analysis (DISP)	RRTs / LVDTs
Leaf has to be fixed with 5 or more weights in a focal plane?	yes	yes	yes	no
Leaf has to be fixed in line with the linear displacement transducer with one weight	no	no	no	yes
A counterweight has to be fixed to the opposite side of the plant?	yes	yes	yes	yes
Artificial landmarks have to be applied?	One dark marker has to be applied on the transition from leaf lamina to petiole to allow clean segmentation excluding petiole	At least four to five artificial landmarks have to be applied	Artificial landmarks have to be applied in form of mini ink droplets or charcoal applied to the leaf surface only if structures on the leaf are too small for instant DISP analysis	no
Strings with low infrared reflectivity have to be used for attachment of weights to the leaves?	yes	no	no	no
Additional illumination with infrared LED clusters is necessary to allow continuous growth analysis during night and day?	yes	yes	yes	no
